# A web-based GPS system for displacement monitoring and failure mechanism analysis of reservoir landslide

**DOI:** 10.1038/s41598-017-17507-7

**Published:** 2017-12-07

**Authors:** Yuanyao Li, Jinsong Huang, Shui-Hua Jiang, Faming Huang, Zhilu Chang

**Affiliations:** 1 Geological Survey, China University of Geosciences, Wuhan, 430074 China; 2 School of Civil Engineering and Architecture, Nanchang University, Nanchang, 330000 China; 3 ARC Centre of Excellence for Geotechnical Science and Engineering, University of Newcastle, NSW, Australia

## Abstract

It is important to monitor the displacement time series and to explore the failure mechanism of reservoir landslide for early warning. Traditionally, it is a challenge to monitor the landslide displacements real-timely and automatically. Globe Position System (GPS) is considered as the best real-time monitoring technology, however, the accuracies of the landslide displacements monitored by GPS are not assessed effectively. A web-based GPS system is developed to monitor the landslide displacements real-timely and automatically in this study. And the discrete wavelet transform (DWT) is proposed to assess the accuracy of the GPS monitoring displacements. Wangmiao landslide in Three Gorges Reservoir area in China is used as case study. The results show that the web-based GPS system has advantages of high precision, real-time, remote control and automation for landslide monitoring; the Root Mean Square Errors of the monitoring landslide displacements are less than 5 mm. Meanwhile, the results also show that a rapidly falling reservoir water level can trigger the reactivation of Wangmiao landslide. Heavy rainfall is also an important factor, but not a crucial component.

## Introduction

Reservoir landslides are serious geological hazards in Three Gorges Reservoir area (TGRA)^1–3^. The local residents and ecological environments are threatened heavily by the reservoir landslide displacements^4,5^. Landslide displacement time series is generally described as a complex non-linear natural phenomenon^6,7^. It is significant to monitor the displacements and to explore the failure mechanism of reservoir landslides.

In recent years, many earth observation techniques have been proposed to monitor landslide displacements, such as precise geodetic measurement^8^, GPS^9–11^, inclinometer^12^, Interferometric Synthetic Aperture Radar (InSAR)^13–15^. Generally speaking, most of these technologies are efficient to monitor landslide displacements. However, there are some limitations in the applications of these technologies. The precise geodetic measurement has disadvantages of heavy workload, poor timeliness and complex working conditions^16^. Inclinometers can only monitor the small deep displacements of landslide^17^. It is difficult for the InSAR to monitor the landslide covered by luxuriant vegetation, and it cannot monitor the landslide displacements real-timely^18^. Comparing to the three techniques, GPS has advantages of high monitoring precision, convenient operation and low cost^19,20^. It has been widely applied to monitor landslide displacements since 1990s^21,22^.

Unfortunately, it is difficult for the conventional GPS technology to monitor the landslide displacements real-timely and automatically. Meanwhile, it is meaningful to build an effective monitoring system which can continuously collect, transfer and storage the landslide displacements, along with the trigger factors in the most significant areas^12,23^. Therefore, in this study, a web-based GPS system is proposed to monitor the landslide displacement time series and its trigger factors^24^. The web-based GPS system provides several advantages: 1) it is based on a real-time monitoring data transport network among sensors, and based on an automatic data processing software^25^; 2) landslide displacements and trigger factors are collected from different data sources and matched on a single server-side framework to avoid complex data management; 3) a remote visual interface in a browser allows the decision-makers to interact with an easy-to-use support system to manage the instruments, and to publish early warning information^12,26^.

In addition, it is necessary to assess the accuracy of the web-based GPS monitoring displacements. The displacement errors mainly contain system and random errors, most of the system errors have been removed in the phase of GPS signals processing. Therefore, the errors of the GPS monitoring displacements are mainly presented as random errors with few system residual errors^24^. Recently, many accuracy assessment methods have been used, such as Kalman filter^27^, smoothing method^28^, and Square-Root Information filter^29^. However, these methods are difficult to effectively extract the random errors from the original GPS monitoring displacements. In this study, the wavelet analysis (WA) is proposed to assess the accuracy of the GPS monitoring landslide displacements^30^. The WA method is developed based on the Fourier transform, however, the WA is superior to Fourier transform both in time and frequency domain for time series processing^31^. There are several types of WA method, such as continuous wavelet transform (CWT), discrete parameter wavelet transform (DPWT), and discrete wavelet transform (DWT)^32^. In this study, the landslide displacements are discrete time series. Hence, the DWT is appropriate for accuracy assessment of discrete landslide displacement, while the CWT and DPWT will generate information redundancy when decomposing the discrete landslide displacements^33^. The DWT method has excellent ability of extracting noisy information from the original GPS monitoring displacements and retaining the characteristics of the original GPS monitoring displacements^34^. The random errors are considered as the noisy information of the original GPS monitoring displacements.

The proposed web-based GPS system is tested on the Wangmiao landslide in TGRA. The accuracy of the GPS monitoring displacements is determined by the DWT method. And the correlation between the GPS monitoring displacements and trigger factors are discussed through qualitative analysis and grey relational degree analysis (GRA). Finally, the failure mechanism of the reservoir landslide is also explored.

## Study Area

The Wangmiao landslide is a typical reservoir landslide, which has been reactivated since the impoundment of Three Gorges Reservoir. There are continuous displacement processes in Wangmiao landslide. This landslide is selected as study area, the real-time landslide displacements and other relative data are monitored using the web-based GPS system.

### Wangmiao landslide in Three Gorges Dam Reservoir, China

#### Natural environmental conditions

**Figure 1:**
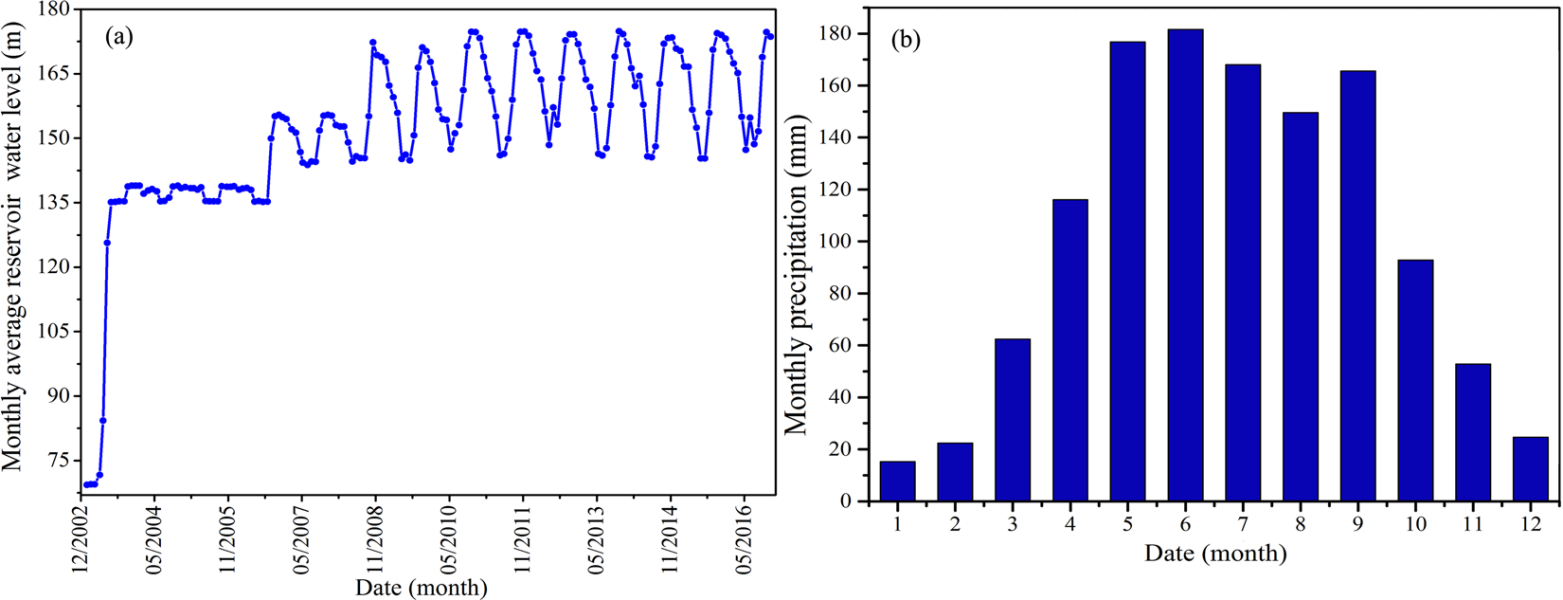
(**a**) Multi-year average reservoir water level and (**b**) monthly average monthly precipitation.

Wangmiao landslide is located on the south bank of the Yangtze River, it is 262 km from the Three Gorges Dam. The impoundment of the Three Gorges reservoir is finished through the following four phases. The original water level was approximately 70 m above sea level during the first phase. The water level fluctuated between 135 m and 140 m during this second phase, which lasted from June 2003 to September 2006. The water level periodically fluctuated between 145 m and 156 m during the third phase, which occurred from October 2006 to September 2008. The water level fluctuated between 145 m and 175 m during the final phase, which occurred from 2009 to 2017 as shown in Fig. 1(a). The Wangmiao landslide is located within the subtropical climate belt, which receives frequent heavy rainfall events during the summer season as shown in Fig. 1(b). The annual average precipitation was 1204 mm between 1950 s and 2016 s.

#### Geological conditions

**Figure 2:**
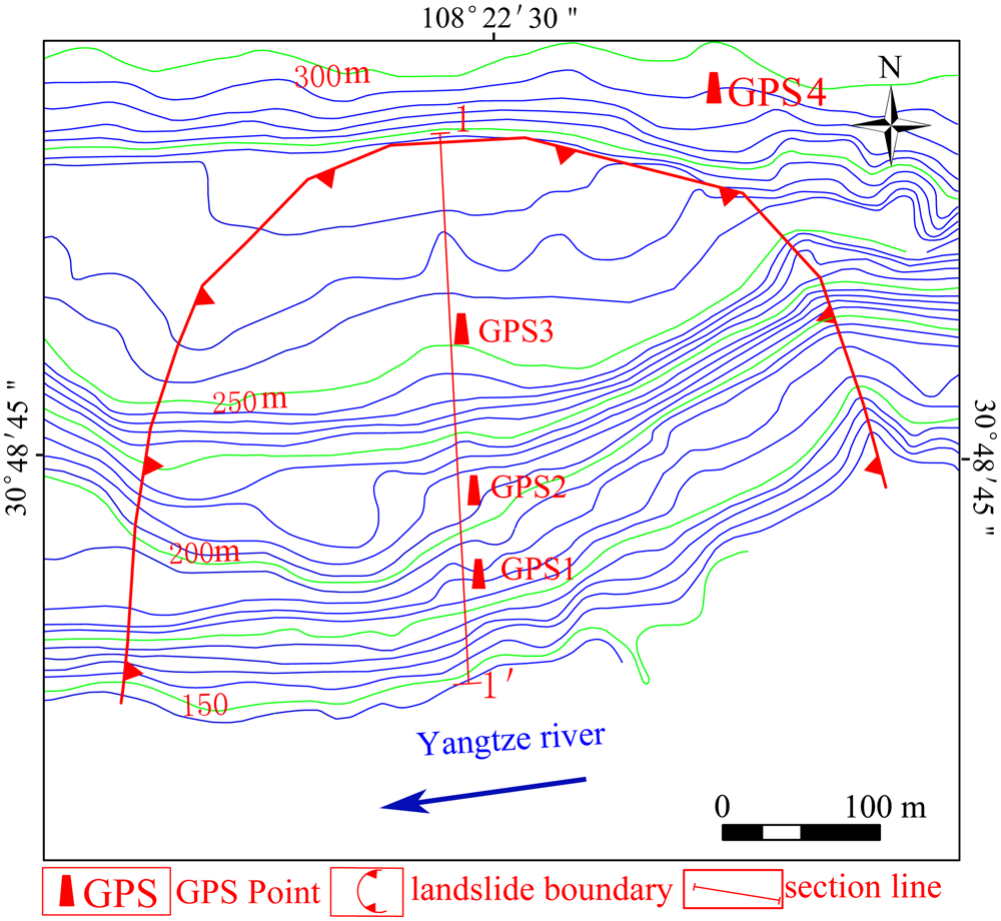
Geographical location map of the Wangmiao landslide (AUTOCAD 2014, https://www.autodesk.com.cn/, drawn by Faming Huang).

The terrain type of Wangmiao landslide is belong to valley hilly topography with eroded accumulation. The landslide is bush-shaped in the plane with a maximum length of 300 m and width of 400 m. The landslide has a mean depth of the sliding surface of approximately 23 m. Hence, the landslide has an volume of 276 × 10^4^ m^3^. The upper boundary of the landslide is defined by the steep slope. The left and right boundaries of the landslide are defined by bedrock and a gully, respectively. The main sliding direction of the landslide is oriented at S 28° E. The topographical map with the monitoring network is shown in Fig. 2.

**Figure 3:**
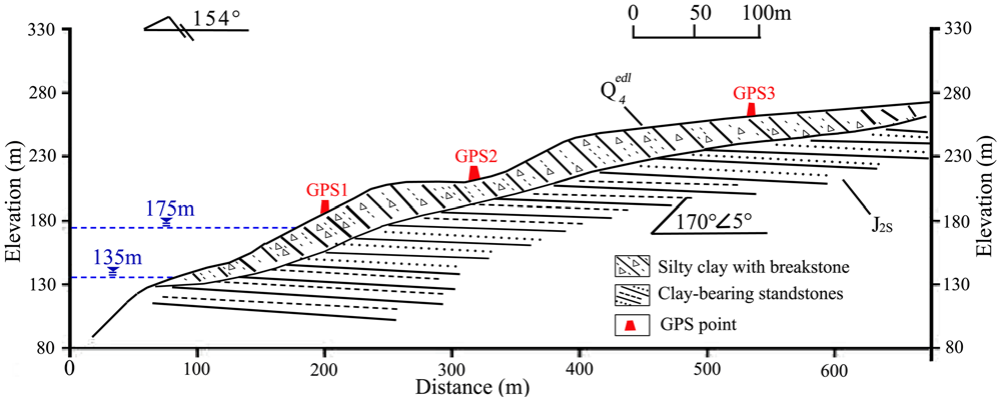
1–1′ geological section of Wangmiao landslide.

The 1-1′ geological section of Wangmiao landslide is shown in Fig. 3. The slope of Wangmiao landslide varies from 5° to 25°. The materials of this landslide are composed of quaternary deposits with fragmented rubbles. The engineering structure of the slide mass is disordered, and the saturated permeability coefficient of the slide mass is small with value of 1.16 × 10^−6^ m/s. The formation lithology of the landslide is thick and bedded in clastic sediments with siltstone and sandstone. The dip direction and dip angle of the formation lithology are 170° and 5°, respectively. The sliding zone is composed of hydrophilic materials such as slit clay and montmorillonite.

#### Deformation characteristics

**Figure 4:**
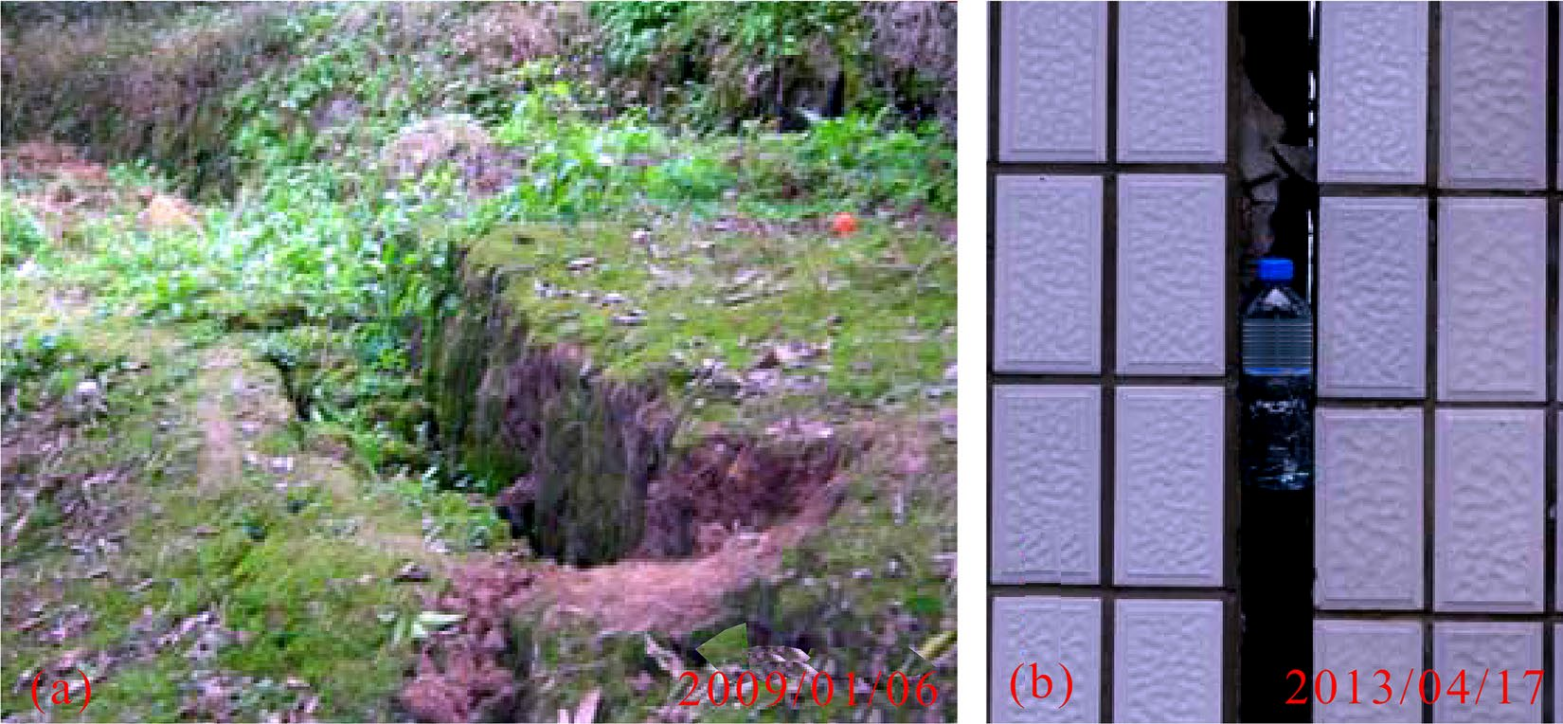
Deformation characteristics of Wangmiao landslide.

The Wangmiao landslide has exhibited local deformation and failure since 2005. Surface investigations have shown that there were shearing and crush-pressing cracks on the landslide. Figure 4(a) shows several surface cracks that occurred under fruit trees, with a maximum crack width of approximately 860 mm. Since 2009, numerous buildings above the study area have been cracked, with crack widths of 5 cm to 40 cm. The buildings have been gradually dismantled. Figure 4(b) shows a tensile crack that occurred in one of the buildings. The width of the tensile crack is 12 cm. Both human lives and properties have been seriously threatened throughout these events. Therefore, it is necessary to monitor the landslide displacement effectively.

### Landslide displacement monitoring using web-based GPS system

**Figure 5:**
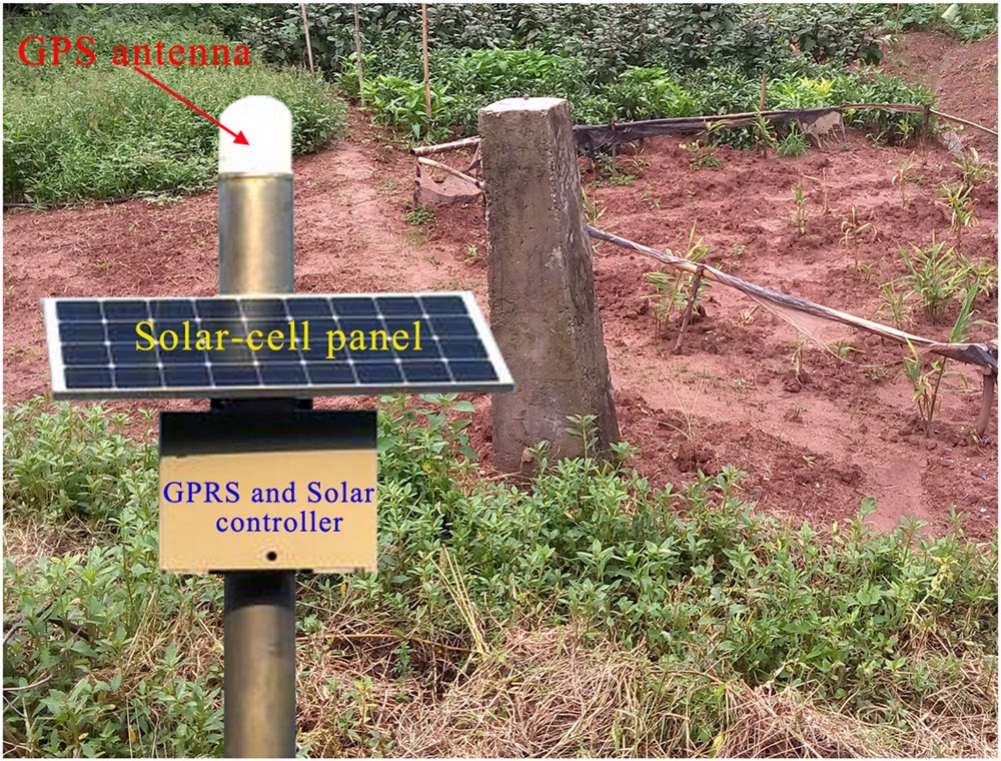
GPS reference and measuring points on Wangmiao landslide.

In order to monitor the displacements of Wangmiao landslide, a web-based GPS system was installed on the landslide on 1 July 2015 as shown in Fig. 5. The web-based GPS system is composed of hardware and software. The hardware mainly includes computer server, GPS antennas, solar-cell panel, GPRS controller, Solar controller, rechargeable battery and other assistant equipment. In this study, a GPS antenna was in a stable zone outside the landslide as a reference point and three other GPS antennas were placed on the landslide as measuring points. The GPS 1, GPS 2 and GPS 3 were respectively located on the frontal, middle and upper part of the landslide as shown in the Fig. 2. The production type of the GPS antenna is HUACE × 300 M with double-frequency signal, the standard of the solar-cell panel is 250 *W*, the standard of the rechargeable battery is 250 *Ah*. In addition, the circuit design of the GPS antenna is of excellent quality, and the GPS antennas have been installed on a place with good environment. Hence, the multipath effect^35^ has been addressed well in this study, and the performance of the GPS monitoring landslide displacements has not been significantly reduced by multipath effect.

**Figure 6:**
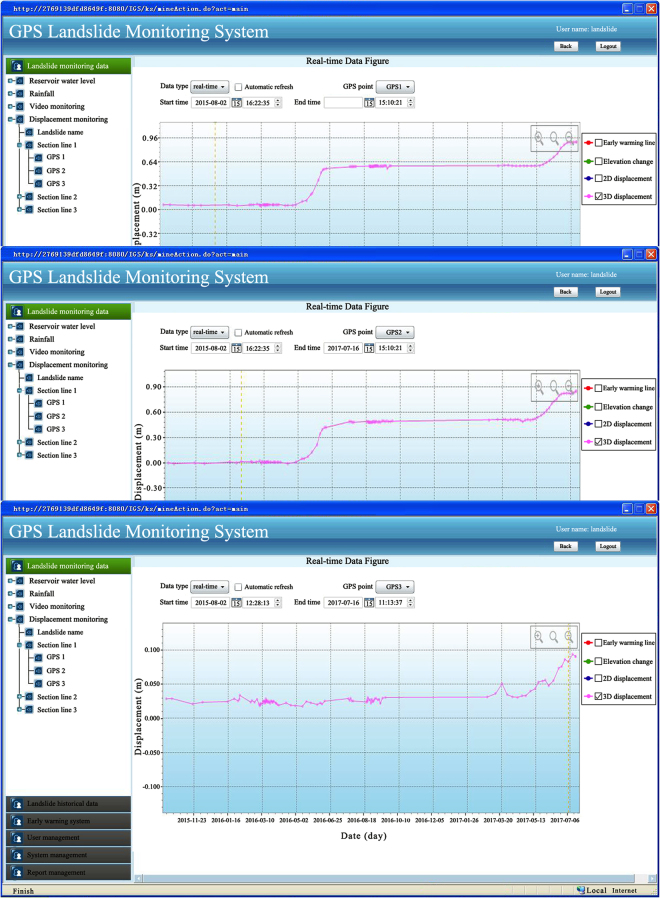
GPS 1, GPS 2 and GPS 3 monitoring landslide displacements.

The software mainly includes data processing, data transformation and display software. The GPS signals are transformed into the data processing software, and the GPS monitoring displacements are stored in the computer server. The HC monitor is used in this study as data processing software^36^. The HCMAS software based on the mixed-architecture of B/S and C/S is used as the monitoring results display software. The HCMAS software can manage, on-line publish and analysis the monitoring landslide displacements and other relative data as shown in Fig. 6.

## Results and Discussion

### Displacements monitored using web-based GPS system

The GPS monitoring displacements of Wangmiao landslide are shown in Fig. 6. It can be seen from Fig. 6 that the displacements of GPS 1 and GPS 2 reach about 938 mm and 868 mm respectively from 1, Aug 2015 to 16, July 2017. However, the displacement of the GPS 3 is about 77 mm. It illustrates that the frontal part of the study area is still in the condition of continuous deformation and it also indicates that the Wangmiao landslide is a pull-type landslide.

### Accuracy assessment of web-based GPS monitoring displacement

**Figure 7:**
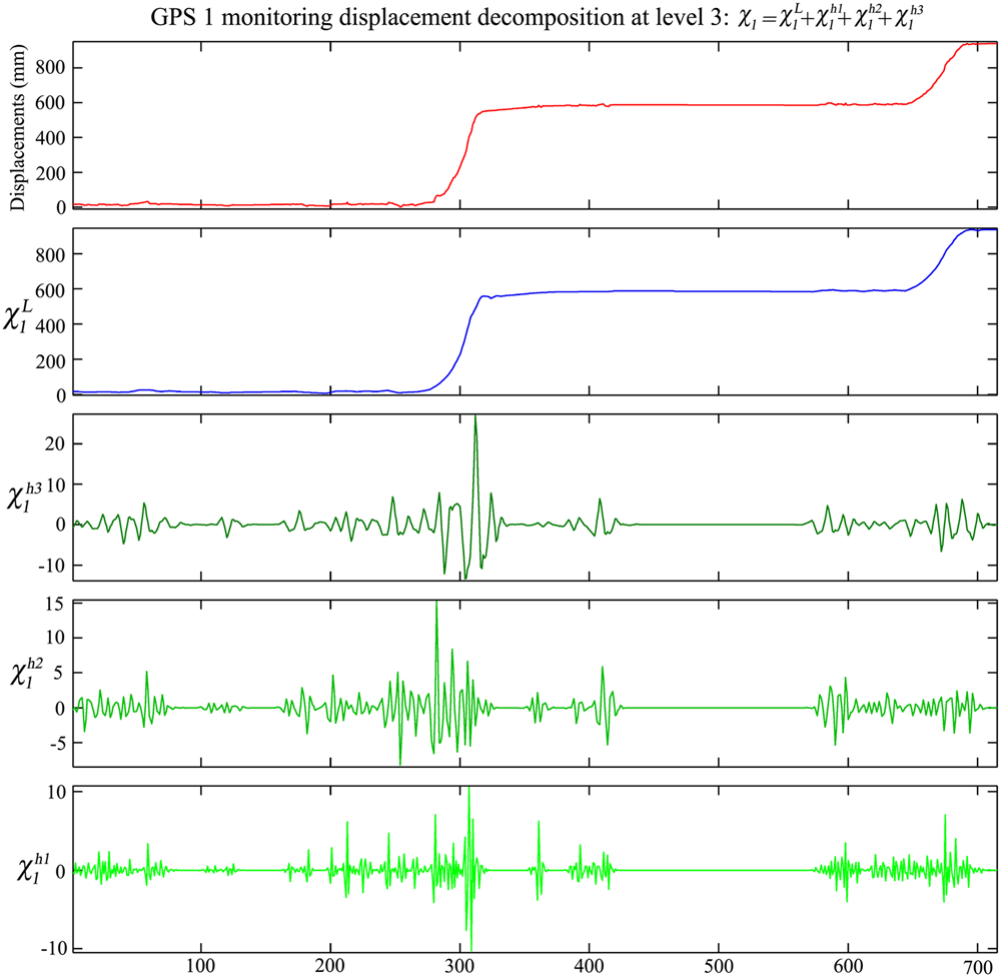
Time series decomposition of landslide displacements on GPS 1.

The DWT method is used to assess the accuracies of web-based GPS monitoring displacements. The decomposition levels of the original displacements of GPS 1, GPS 2 and GPS 3 are set to three according to the Equation (5). The *db*3 wavelet function is used in this study to decompose the displacements. Hence, the original displacements of GPS 1, GPS 2 and GPS 3 are respectively decomposed into one low-frequency component and three high-frequency components. Taking the displacement of GPS 1 as example, the original GPS monitoring displacement is decomposed into four time series with different frequencies as shown in Fig. 7.

**Figure 8:**
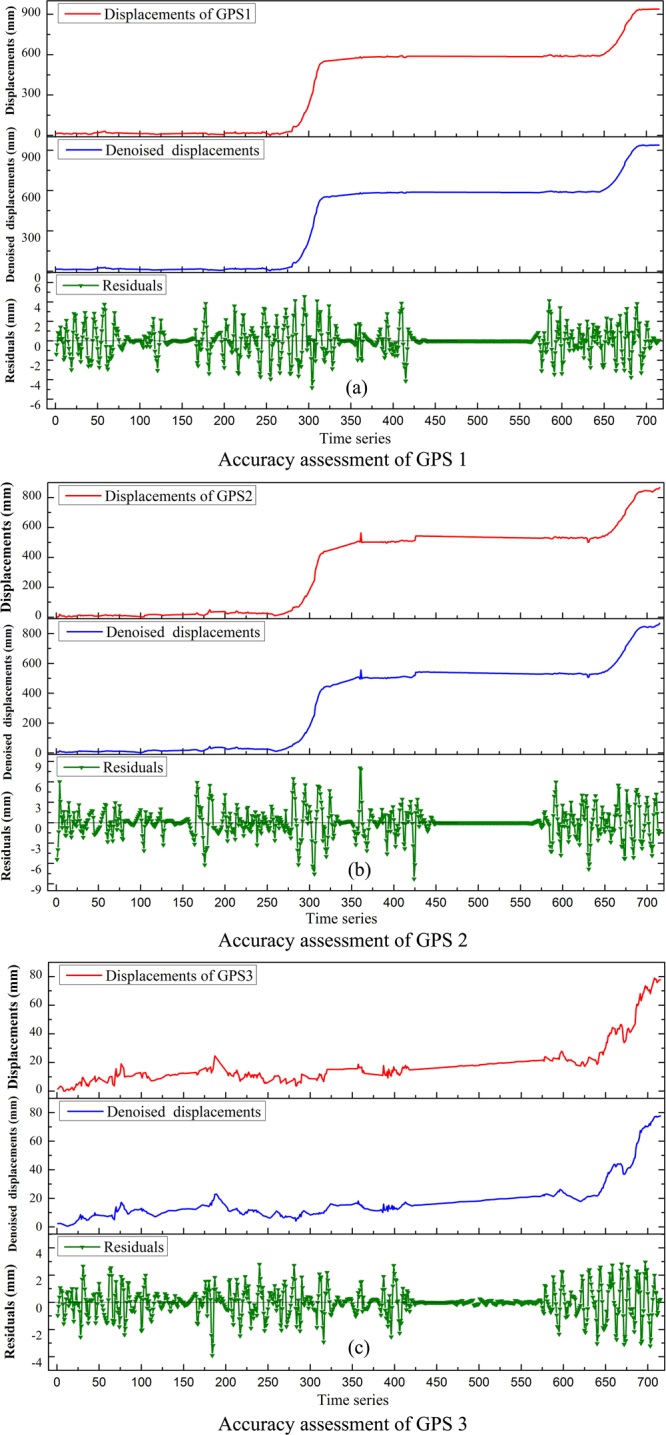
Accuracy assessment of GPS 1 (**a**), GPS 2 (**b**) and GPS 3 (**c**) monitoring displacements using DWT method.

Note that the errors of the web-based GPS system are mainly contained in the three high-frequency components. In this study, the threshold values of wavelet coefficients of each high-frequency component are determined by the soft threshold method. The non-white noise is selected as the noise structure^37^. The threshold values of the first, second and third high-frequency component of GPS 1 displacement are 5.556, 3.015 and 0.839, respectively, according to Equation (6). The threshold values of the first, second and third high-frequency component of GPS 2 displacement are 11.112, 3.775 and 1.194, respectively. And the threshold values of GPS 3 displacement are 5.376, 2.606 and 0.711, respectively. Finally, the de-noised displacements and random errors of the web-based GPS system are obtained through inverse wavelet transform as shown in Fig. 8. In addition, the random errors of the displacements monitored by the web-based GPS system are shown in Table 1. It can be seen from Table 1 that the *RMSEs* of the three displacements are all less than 3 mm, which indicates that the monitoring accuracies of the web-based GPS system are satisfied.

**Table 1:** Errors of landslide displacements monitored by the web-based GPS system.

	*RMSE* (mm)	*MAE* (mm)	Maximum (mm)	Minimum (mm)
GPS 1	1.413	0.928	4.557	−4.824
GPS 2	2.048	1.337	8.055	−8.280
GPS 3	1.022	0.694	2.957	−3.905

### Failure mechanism analysis of landslide

#### Time series analysis of landslide displacement

**Figure 9:**
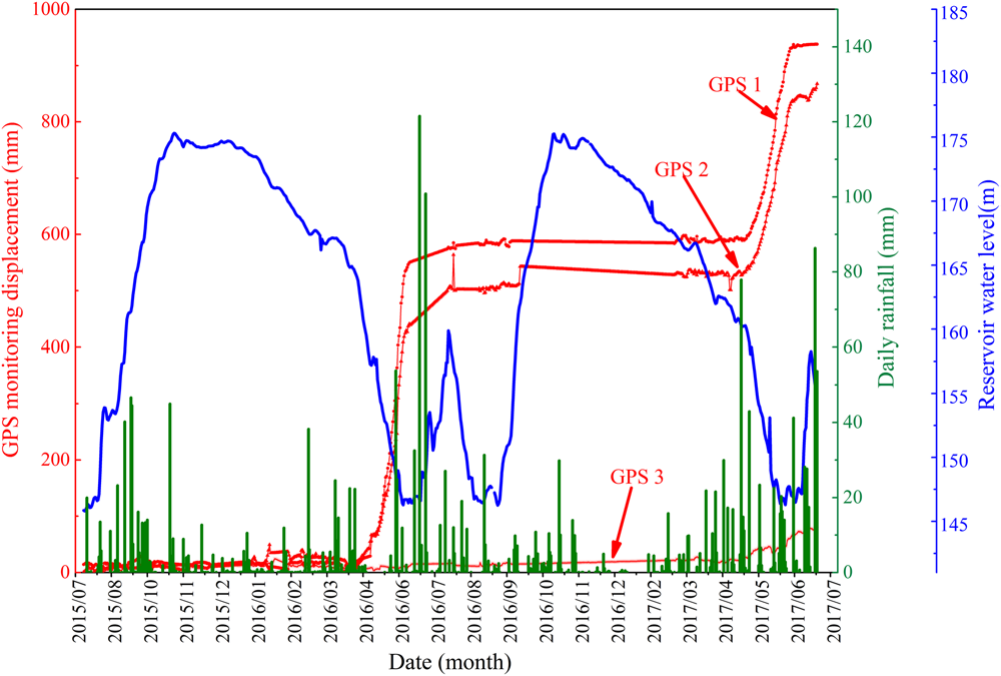
Correlation of landslide displacements, daily rainfall and reservoir water level.

As shown in Fig. 9 that Wangmiao landslide has been divided into three parts: front, middle and upper parts. The displacement processes of the landslide have developed progressively from the front part to the middle part. The upper part of the landslide is only deformed slightly from 2, Aug 2015 to 29, April 2017, then there are great deformation values from 29, April 2017 to 16, July 2017. The space needed for the middle part to slide slowly is supplied by the failure of the front part. It can also be seen from Fig. 9 that there are step-like characteristics in the landslide displacements on GPS 1 in the front part and GPS 2 in the middle part. The displacement characteristics of the landslides are affected by the seasonal and periodic trigger factors.

The fluctuations of the reservoir water level and heavy rainfall could be the trigger factors of the landslide failure as shown in Fig. 9. The deformation cycles of the landslide from 2, Aug 2015 to 16, July 2017 can be divided into two parts according to the seasonal fluctuation of the reservoir water level and heavy rainfall. The first deformation cycle is from 2, Aug 2015 to 16, Aug 2016. The deformation values are both about 25 mm when the reservoir water level is in the stage of rising with abundant rainfall from 2, Aug 2015 to 1, Nov 2015. Then the deformation values are still small when the reservoir water level is in the stage of high-level with small rainfall from 2, Nov 2015 to 24, April 2016. But There are sudden severe deformations when the reservoir water level is in the stage of falling with abundant rainfall from 25, April 2016 to 23,July 2016. And then the deformation values become small again when the reservoir water level is in the stage of rising with gradually reduced rainfall from 24,July 2016 to 17, Aug 2016.

Another landslide deformation cycle can be found in the GPS 1 and GPS 2 monitoring displacements from 17, Aug 2016 to 16, July 2017. There are almost the same deformation laws between the first and second deformation cycles. It can be seen from the two deformation cycles that the fast falling of reservoir water level is the main factor resulting in the severe deformations, while the heavy rainfall is to further accelerate the deformation of Wangmiao landslide.

#### Relational degree analysis between landslide displacements and trigger factors

It is difficult to calculate the quantitative correlation between the landslide displacements and trigger factors for the whole web-based GPS monitoring period, because the landslide deformation system is composed of several different deformation parts according to the seasonal changes of trigger factors. Hence, the landslide displacements, the daily average reservoir water level and daily rainfall time series from 19, April 2016 to 10, July 2016 are selected as typical cases to calculate the quantitative correlation analysis in this study.

The landslide displacements are set as reference time series, the daily average reservoir water level and daily rainfall are set as comparative time series. The GRA is applied to calculate the non-linear correlation between the reference and comparative time series according to the section 2.3. The results show that the GRA values of reservoir water level on landslide displacements of GPS-1, GPS-2 and GPS-3 are 0.786, 0.763 and 0.728, respectively. And the GRA values of rainfall on landslide displacements of GPS-1, GPS-2 and GPS-3 are 0.598, 0.591 and 0.541, respectively. The above GRA values suggest that, the reservoir water level and rainfall have important effects on the landslide displacements, and the reservoir water level has higher influence than the rainfall. Meanwhile, the results of GRA analysis are agree with the qualitative analysis in section 4.3.1.

#### Landslide failure mechanism analysis

The reactivation of the Wangmiao landslide is controlled by its geological conditions as described in section 3.1. The materials of the slide mass are characterised by a loose structure and low mechanical strength. The sliding zone of the landslide is hydro-expansiveness, and the anti-slide force of the landslide declines under the immersion of the water. Meanwhile, the freed surface is another passive factor for landslide displacements.

The results in section 4.3.1 and the grey relational degree analysis show that the main external cause of landslide displacements is the decrease of rapid reservoir water level to a minimum level. The Wangmiao landslide can be regarded as a hydrodynamic pressure landslide because its saturated permeability coefficient is small and its displacement process is sensitive to the drawdown of reservoir water level from 175 m to 145 m. There is water head difference between the landslide groundwater level and the reservoir water level when the reservoir water level fluctuates. The reverse hydrodynamic pressure formed by the water head difference accelerates the landslide deformation when the reservoir water level decreases. On the contrary, the normal hydrodynamic pressure formed by the water head difference prevents the landslide deformation when the reservoir water level rises. Meanwhile, the hydrostatic pressure and the hydraulic uplift pressure on the landslide also result in landslide deformation when the reservoir water level decreases; while the hydrostatic pressure and the hydraulic uplift pressure prevent landslide deformation when the reservoir water level rises.

Heavy rainfall also accelerates the landslide deformation. The northern portion of the study area undergoes erosion and is saturated due to the reservoir water and heavy rainfall^38^. These processes decrease the matric suction of the rock-soil body and affected the slip zone. Additionally, the unit weight and seepage pressure of the slide body will continue to increase under the condition of rainfall infiltration. However, the rainfall is not the crucial trigger factor of landslide deformation. It is because that the permeability coefficient of the landslide is small, most of the rainfall flows to the Yangtze river from the slope and the gullies, only a small amount of rainfall percolates into the slide mass.

#### Future prediction of landslide displacements

It is very difficult to do quantitative prediction of the landslide displacements. One important reason is that the landslide displacements are chaotic time series containing stochastic and deterministic components. Another reason is that the obtained landslide displacements are not long enough to build the prediction model. Furthermore, it is complex to train and test the mathematical prediction model.

**Figure 10:**
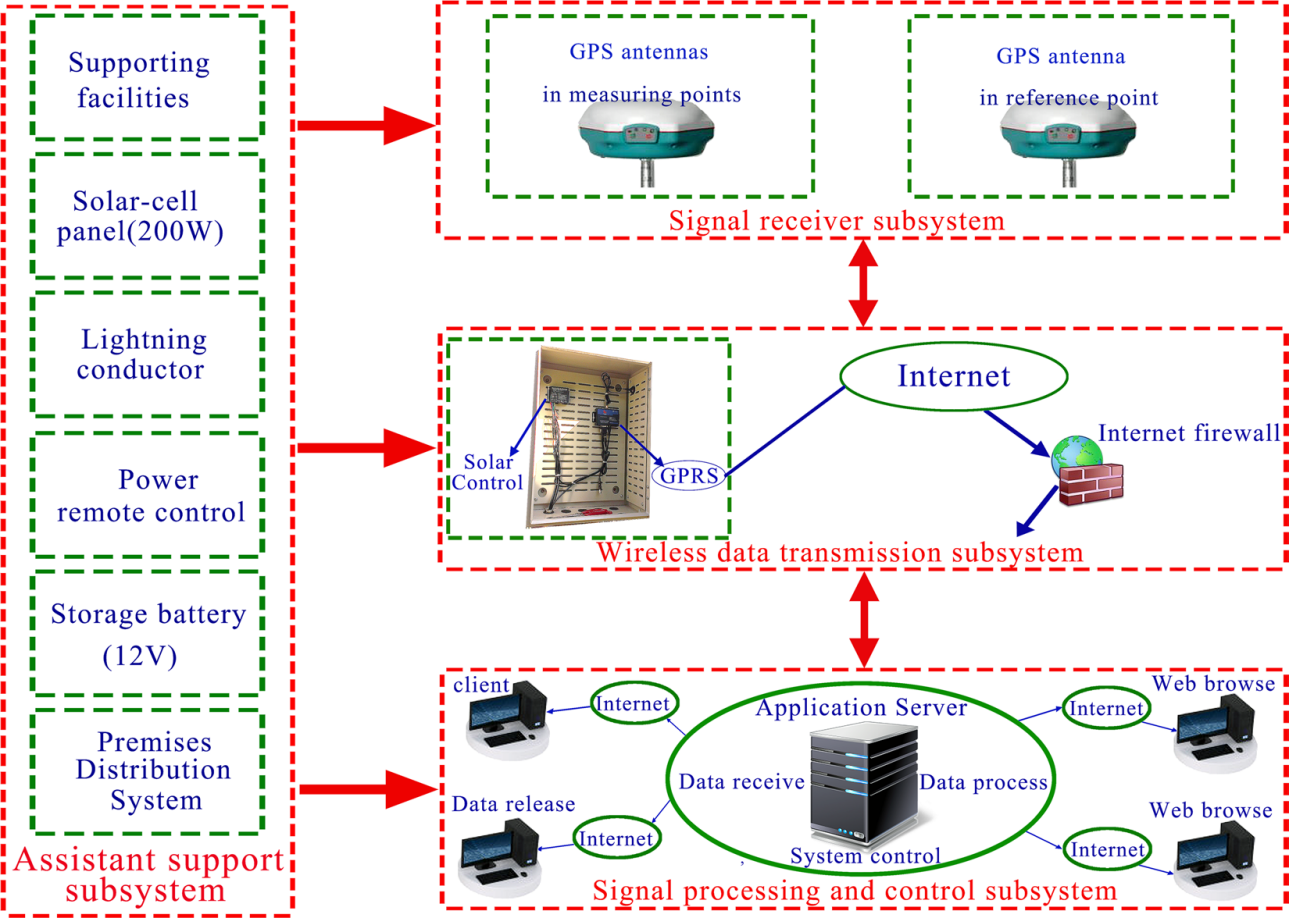
General architecture of web-based GPS system (Drawn by Faming Huang).

Fortunately, qualitative predictions of landslide displacements during the sudden severe deformation period can be carried out. It can be seen from Fig. 10 that the sudden severe landslide displacements of GPS-1, GPS-2 and GPS-3 from 19, April 2016 to 10, July 2016 are 554 mm, 469 mm and 5 mm, respectively. And the landslide displacements of GPS-1, GPS-2 and GPS-3 from 19, April 2017 to 10, July 2017 are 348 mm, 318 mm and 56.5 mm, respectively, Hence, it can be deduced that, under the similar conditions of fluctuation of reservoir water level and rainfall, the landslide displacements of GPS-1 will vary between 348 mm and 554 mm, GPS-2 will vary between 318 and 469 mm, and GPS-3 will vary between 5 mm and 56.5 mm from 19, April 2018 to 10, July 2018.

## Conclusion

This study proposes a web-based GPS system to monitor the displacements and to study the failure mechanism of the Wangmiao landslide in TGRA. The monitoring accuracies of the landslide displacements are assessed using the DWT method. The correlation between the reservoir water level fluctuation, rainfall and GPS monitoring landslide displacements are analyzed through qualitative analysis and grey relational degree analysis (GRA). The results show that the web-based GPS system can precisely, real-timely and automatically monitor the landslide displacements through remote control. And the results also show that the geological conditions control the landslide instability, while the decrease of rapid reservoir water level triggers the landslide deformation. Heavy rainfall was also an important factor, but not a crucial component.

## Methods

### Introduction and constitution of the web-based GPS system

There are several GPS positioning principles for landslide displacement monitoring, such as pseudo range measurement^39^, carrier phase measurement^40^ and static relative positioning measurement^41^. In this study, the static relative positioning measurement with millimeter accuracy is adapted in the web-based GPS system, because it can eliminate the orbit and atmosphere errors by a spatial correlation between the reference point and measuring points^25^. The static relative position measurement can also remove the errors between the clocks in the satellites and GPS receivers. It is because that the errors of orbit and atmosphere are connected with the distance between the GPS reference point and measuring point. Therefore, a GPS antenna should be placed near the target landslide as a certain reference point, some other GPS antennas should be placed on the landslide as measuring points. The three-dimensional coordinate values of all the points are measured, then the three-dimensional coordinate values of the measuring points are compared to the reference point to determine the landslide displacements of the measuring points.

The proposed web-based GPS system is composed of signal receiver, wireless data transmission, signal processing and control, and assistant support subsystems. Its architecture is shown in Fig. 10. The GPS positioning signals are acquisitioned in the signal receiver subsystem, at least five satellite signals should be received at the same time by the GPS antennas, then the received signals will be transmitted to the signal processing and control subsystem through the wireless data transmission subsystem in real-time. The GPS reference and measuring points record eight-hours signals synchronously, this process is known as a monitoring phase. In the signal processing and control subsystem, the landslide displacements based on the World Geodetic System-84 (WGS-84) can be obtained.

#### Signal receiver subsystem

Signal receiver subsystem is mainly composed of GPS antennas, which should be placed without GPS signals obstructed by buildings or trees. The GPS antennas are put on the reference point and measuring points, respectively. The positioning signals are received through GPS antenna, and then are interpreted into messages in binary format by GPS receiver card. The signals contain carrier phase, pseudo-range, the known coordinate and some other data^42^. The reference and measuring points receive signals synchronously. Meanwhile, the interpreted messages are transmitted into the signal processing and control subsystem.

#### Data transmission subsystem

The data transmission subsystem contains the General Packet Radio Service (GPRS) and Internet network^43^. The GPRS is a type of wireless packet switching technology based on global system for mobile communications. It can provide Internet Protocol (IP) connection from end to end. Therefore, the GPRS is able to provide all the similar functionality as the Internet network. The GPRS wireless module is used to connect the received GPS signals with the signal processing and control subsystem real-timely and steadily. In addition, the rainfall and reservoir water levels are also transmitted into the signal processing and control subsystem through the GPRS.

#### Signal processing and control subsystem

The signal processing and control subsystem is composed of computer server, GPS signal processing software (HC monitor^36^), remote client terminal and display software. In the computer server, signal difference processing method is applied to obtain *mm* level positioning results of measuring points using GPS signal processing software in real time. The relative displacements between the reference point and the measuring points are calculated in real time using base-line solution method in computer server^44^. Then the monitoring landslide displacements are stored in database and displayed. Therefore, it is able to obtain the real-time landslide displacements at any time in the remote client terminal. And it becomes possible for geo-researchers and environmental departments to remotely access landslide displacements from anywhere at any time.

#### Assistant support subsystem

The assistant support subsystem is composed of equipment cabinet, lightening conductor, storage battery and solar-cell panel^26^. The equipment cabinet is used for installing solar energy controller and GPRS controller. The storage battery is applied to save the abundant electricity from solar-cell panel and to provide a stable power. The solar-cell panel is aimed to accept the sun light and to convert the sun light to electricity.

### Accuracy assessment of displacements using DWT method

#### DWT method

DWT method^32^ is a multi-frequency analysis tool which can decompose non-linear and non-stationary time series into several stationary time series from both time and frequency domains. The mother wavelet function *φ*(*t*) can be defined as:

1 $${\int }_{-\infty }^{+\infty }\phi (t)dt=0$$

2 $${\phi }_{a,b}(t)={|a|}^{-\frac{1}{2}}\phi (\frac{t-b}{a})$$

where *φ*_*α,b*_(*t*) is a successive wavelet, α is frequency factor, *b* is a time factor, α and *b* are real numbers. The wavelet transform is a function of variables *a* and *b*. *a* can be interpreted as a dilation or contraction factor, *b* can be interpreted as a temporal translation of the function *φ*(*t*). For a discrete time series *f*(*t*), when *f*(*t*) occurs at a different time *t*, the DWT can be defined as:

3 $${W}_{f}(u,v)={2}^{-\frac{u}{2}}\sum _{t=0}^{K-1}f(t){\phi }^{\ast }({2}^{-u}i-v)$$

where *W*_*f*_(*u*, *v*) is the wavelet coefficient of a discrete wavelet with scale α = 2^*u*^ and location *b* = 2^*u*^ *v*; *f*(*t*) is a finite time series, where $t=0,1,2,\cdots \cdots ,K-1$; *K* is an integer power of 2 (*K* = 2^*U*^); and *v* the time translation parameter, which varies from 0 < *v* < 2^*U*−*u*^1, where 1 < *u* < *U*. There are two sets of functions named high-pass filters and low-pass filters in the DWT method. The discrete time series can be decomposed into low-frequency time series through the low-pass filters and high-frequency time series through the high-pass filters. The wavelet coefficients *W*_*u*_(*t*), where $u=1,2,\cdots \cdots ,U$, can capture detailed characteristics of interpreted values in the time series. The residual term $\bar{W}(t)$ captures the approximated characteristics of the time series. Therefore, the trend, period and detailed characteristics of a landslide displacement time series can be effectively extracted through DWT method.

Meanwhile, the Mallat algorithm^45^ is used in the DWT method to decompose the GPS monitoring landslide displacement *x*_*p,i*_ into a low-frequency component and several high-frequency components at limit levels as:

4 $${x}_{p,i}={x}_{p,i}^{l}+{x}_{p,i}^{h1}+{x}_{p,i}^{h2}\cdots \cdots +{x}_{p,i}^{hL}$$

where *p* is the serial number of GPS points, *L* is the decomposition level; ${x}_{p,i}^{l}$ is the low-frequency component, which generally suggests the approximate trend in landslide displacement; and ${x}_{p,i}^{h1}\,\,,\,\,\,{x}_{p,i}^{h2}\cdots \cdots \,\,,\,\,{x}_{p,i}^{hL}$ are the first, second, ……, *L*th level high-frequency components, respectively, which mainly reflect the periodic and detailed characteristics of the landslide displacement.

In addition, the Daubechies wavelet (*dbN*) function is selected as an appropriate wavelet function because of its localization capability in both time and frequency domains^46^. It is also very important to select an appropriate number of the decomposition levels. It is determined according to Nourani, Alami and Aminfar^47^, Wang and Ding^48^ as:

5 $$L=Round\,[\mathrm{log}(N),0]$$

where *L* is the decomposition level, *N* is the number of displacement time series.

#### Accuracy assessment using DWT method

The original GPS monitoring landslide displacements are composed of valuable displacements and random errors. It is generally acknowledged that the valuable displacements are mainly presented as low-frequency component, and the random errors are contained in the high-frequency components. Meanwhile, the DWT method can decompose the original landslide displacements into one low-frequency component and several high-frequency components. Therefore, we can extract the random errors from the original landslide displacements using the DWT method:

1. The original landslide displacements are decomposed into one low-frequency and several high-frequency components based on the selected wavelet function and optimal decomposition levels.
2. The wavelet coefficients in each high-frequency component are determined using the threshold values.
3. The valuable displacements are obtained through inverse wavelet transform using wavelet coefficients of modulus maximum.

The most important process for wavelet de-noising is to adjust the wavelet coefficients of the high-frequency components based on threshold rule. The threshold rule is proposed by Donoho^49^ to determine the threshold values for wavelet de-noising of a given signal. The wavelet coefficients of the high frequency components are deleted when their modulus are less than a given threshold, and the remaining coefficients are made use to reconstruct the de-noised displacement time series through taking the inverse wavelet transform. A universal threshold value is proposed by Donoho and Johnstone^50^ as:

6 $${T}_{L}={\sigma }_{L}\sqrt{2\,\mathrm{ln}\,N}$$

where *T*_*L*_ is the variance of the detail coefficients at decompose level *L*, *N* is the number of displacement time series. There are two kinds of threshold methods: the hard threshold and soft threshold method. The soft threshold, a continuous mapping method, pushes all coefficients towards zero, while the hard threshold method forces coefficients to zero or leaves them untouched. The soft threshold method is more popular than hard threshold^51^. The de-noised landslide displacement time series can be reconstructed using the selected wavelet coefficients and scales.

#### Accuracy assessment indexes

The Root Mean Square Error (*RMSE*), Mean Absolute Error (*MAE*), Maximum and Minimum errors are used as accuracy assessment indexes of GPS monitoring displacements^24^. The *RMSE* is calculated as:

7 $$RMSE=\sqrt{\frac{\sum _{i=1}^{N}{({x}_{p,i}-{\bar{x}}_{p,i})}^{2}}{N}}$$

where ${\bar{x}}_{p,i}$ is the average value of the original displacements. The lower the *RMSE* is, the more accurate the GPS monitoring displacements. In addition, the *MAE* is also used to assess the accuracy of the GPS monitoring displacements as:

8 $$MAE=\frac{1}{N}\sum _{i=1}^{N}|{x}_{p,i}-{\bar{x}}_{p,i}|$$

### Grey relational degree analysis

The grey relational degree analysis (GRA) is initially proposed by Deng and J.^52^. It is a mathematical measurement method that calculates the uncertain relations between the reference time series and comparative time series in a given system^53^. Hence, the quantitative description and comparison of the developing tendency of a given system can be described using the GRA method. Basic process of GRA is described as follows:

*Step 1*: Determining the reference time series and comparative time series.

The reference time series reflects the behavior characteristic of the given system, it can be formulated as ${X}_{0}=\{{x}_{j}|\,\,\,\,j=1,\,\,2,\,\,\cdots \cdots ,\,\,n\,\,\,\}$. The comparative time series reflects the trigger factors of the reference time series, it is formulated as:

9 $${X}_{i}=\{{x}_{ij}|\,\,\,\,i=1,\,\,2,\,\,\cdots \cdots ,\,\,n;\,\,\,\,j=1,\,\,2,\,\,\cdots \cdots ,\,\,m\,\,\}$$

where *xij* is the value of *j* indicator in sample *i*; *m* is the number of indicators, *n* is the number of sample.

*Step 2*: The comparative time series is normalized into [0, 1]. It is necessary to standardize the comparative time series because of the different parameters of the original data dimension.

*Step 3*: Calculation of the relational coefficient. The formula for calculating the relational coefficient of normalized comparative time series is shown as:

10 $${\zeta }_{ij}=\frac{{{\rm{\Delta }}}_{\min }+\lambda {{\rm{\Delta }}}_{\max }}{{{\rm{\Delta }}}_{ij}+\lambda {{\rm{\Delta }}}_{\max }}$$

where *ζ*_*ij*_ is the calculated relational coefficient, ${{\rm{\Delta }}}_{\min }=\mathop{\min }\limits_{i}\mathop{\min }\limits_{j}{{\rm{\Delta }}}_{ij}$, ${{\rm{\Delta }}}_{\max }=\mathop{\max }\limits_{i}\mathop{\max }\limits_{j}{{\rm{\Delta }}}_{ij}$, ${{\rm{\Delta }}}_{ij}=|{{x}^{^{\prime\prime} }}_{ij}-{{x}^{^{\prime\prime} }}_{j}|$, ${{x}^{^{\prime\prime} }}_{ij}$ and ${{x}^{^{\prime\prime} }}_{j}$ are the results of the standardization of the original data. *λ* ∈ [0, 1] is the resolution ratio, it is usually set to 0.5.

*Step 4:* Determination of grey relational degree. Grey incidence degree judgment matrix can be calculated using formula (11) as:

11 $$G=(\begin{array}{cccc}{\zeta }_{11} & {\zeta }_{12} & \cdots  & {\zeta }_{1m}\\ {\zeta }_{21} & {\zeta }_{22} & \cdots  & {\zeta }_{1m}\\ \vdots  & \vdots  & \vdots  & \vdots \\ {\zeta }_{n1} & {\zeta }_{n2} & \cdots  & {\zeta }_{nm}\end{array})$$

where *ζ*_*ij*_ represents the grey relational degree of the sample *i* to the indicator *j*. It is regarded as the degree of relationship between the factor *j* value and the ideal value of each sample. Therefore, the average value of *ζ*_*ij*_ represents the final grey relational degree of the indicator *j* in the whole indicator space. the final grey relational degree of the indicator *j* is shown as:

12 $${c}_{j}=\frac{1}{n}\sum _{i=1}^{n}{\zeta }_{ij}$$

### Data availability statement

The datasets generated during and/or analysed during the current study are available from the corresponding author on reasonable request.
